# Hepatic Aquaporin 8 Promotes Alcohol Consumption and Ameliorates Alcohol-Induced Liver Injury by Facilitating Acetaldehyde Excretion

**DOI:** 10.7150/ijbs.122713

**Published:** 2026-01-01

**Authors:** Cheng Chen, Yu-Hong Lin, Dechun Feng, Yukun Guan, Yaojie Fu, Yang Wang, Luca Maccioni, Deniz Seyhan, Tiantian Yao, Shoupeng Wei, Li Zhang, George Kunos, Bryan Mackowiak, Bin Gao

**Affiliations:** 1 Laboratory of Liver Diseases, National Institute on Alcohol Abuse and Alcoholism, National Institutes of Health, Bethesda, MD, USA.; 2 Laboratory of Physiologic Studies, National Institute on Alcohol Abuse and Alcoholism, National Institutes of Health, Bethesda, MD, USA.; 3 Laboratory for Integrative Neuroscience, National Institute on Alcohol Abuse and Alcoholism, National Institutes of Health, Bethesda, MD, USA.; 4 Lead contact.

## Abstract

Acetaldehyde (AcH), the first metabolite of ethanol, is an aversive and bioactive compound that plays a key role in modulating alcohol consumption and liver injury. The traditional notion is that AcH is primarily metabolized in the liver by aldehyde dehydrogenase 2 (ALDH2). However, our recent study suggests that the gut-liver ALDH2 axis, rather than the liver alone, plays a key role in metabolizing and clearing AcH partially via bile secretion. Aquaporin 8 (AQP8) is a membrane channel that localizes at the canalicular membrane of hepatocytes and is known to increase bile flow. Here, we identify hepatic AQP8 as an important channel of AcH excretion, mediating its efflux from hepatocytes into bile both with and without altering bile flow. We demonstrated that acute alcohol exposure enhanced AQP8-mediated bile flow and AQP8 promoted hepatic AcH clearance and increased alcohol consumption in both male and female mice. Furthermore, chronic alcohol exposure downregulated hepatic *Aqp8* expression, whereas overexpression of hepatic *Aqp8* alleviated dysregulated lipid metabolism and liver inflammation in a murine model of alcohol-associated liver disease (ALD). Collectively, our study uncovers a novel role for AQP8 in AcH secretion, demonstrating how this pathway influences both alcohol consumption and liver injury. These findings provide a foundation for exploring AcH excretion as a therapeutic target in alcohol use disorder and ALD.

## Introduction

Acetaldehyde (AcH), the primary product of ethanol (EtOH) metabolism, is a known carcinogen whose broad physiological effects are largely dependent on tissue type and concentration 1. Aldehyde dehydrogenase 2 (ALDH2) metabolizes AcH to acetate, and the inactive ALDH2 polymorphism (Aldh2*2), which is prevalent in the Eastern Asian population, causes the accumulation of AcH following alcohol consumption 2. Individuals carrying the Aldh2*2 allele are protected from alcohol use disorder (AUD) due to the aversive effects of AcH accumulation. However, they have higher rates of head & neck and esophageal cancers, likely due to the high level of AcH in the mouth and throat after alcohol consumption 3-5.

Our recent studies have shown that liver and gut ALDH2 work synergistically to clear systemic AcH, with AcH excretion in bile serving as a critical pathway for its clearance via enterohepatic circulation 6, 7. Notably, while ethanol distributes uniformly across tissues according to water content 1, AcH shows disproportionately high concentrations in bile compared to serum in acute alcohol-treated mice 6. This indicates that active transport may play a role in determining the tissue concentration of AcH in addition to ALDH2-mediated metabolism, which deserves further investigation.

A recent study revealed that hepatic aquaporin 8 (AQP8) facilitates bile flow and its impairment contributes to gallstone formation 8. In the liver, AQP8 is localized to the canalicular (apical) membrane of hepatocytes, where it mediates the transport of water and other substrates such as glycerol, ammonia, and hydrogen peroxide 8-10. In general, aquaporins (AQPs), also known as water channels, are ubiquitously expressed in cells to mediate water and water-soluble solute reabsorption and secretion 11, 12. AQPs contribute to various cellular processes and disease progression 13, 14. Given its localization and solute transport capabilities, we hypothesized that AQP8 facilitates AcH excretion into bile, which could, in turn, influence alcohol drinking behavior and the progression of alcohol-associated liver disease (ALD).

In this study, using genetic *Aqp8* mouse models, *in vivo* and *ex vivo* surgical approaches, and primary hepatocyte assays, we demonstrate that AQP8 facilitates hepatic AcH efflux, both dependent and independent of changes in bile flow. We also show that binge ethanol exposure enhances AQP8-mediated bile flow, promoting AcH clearance. In behavioral tests, AQP8 promotes voluntary and high-concentration alcohol consumption in both male and female mice. Notably, chronic alcohol consumption suppresses hepatic *Aqp8* expression, and restoring *Aqp8* levels ameliorates lipid metabolism dysregulation and liver inflammation in ALD. Our study is the first to report on AcH excretion, providing the foundation for understanding AQP8-facilitated AcH excretion from hepatocytes into bile. Modification of AcH excretion further affects AcH clearance and metabolism, contributing to ALD progression and drinking preference. This offers novel therapeutic modalities for the treatment of ALD and AUD.

## Results

### Ethanol increases bile flow through hepatic AQP8

**Figure 1:**
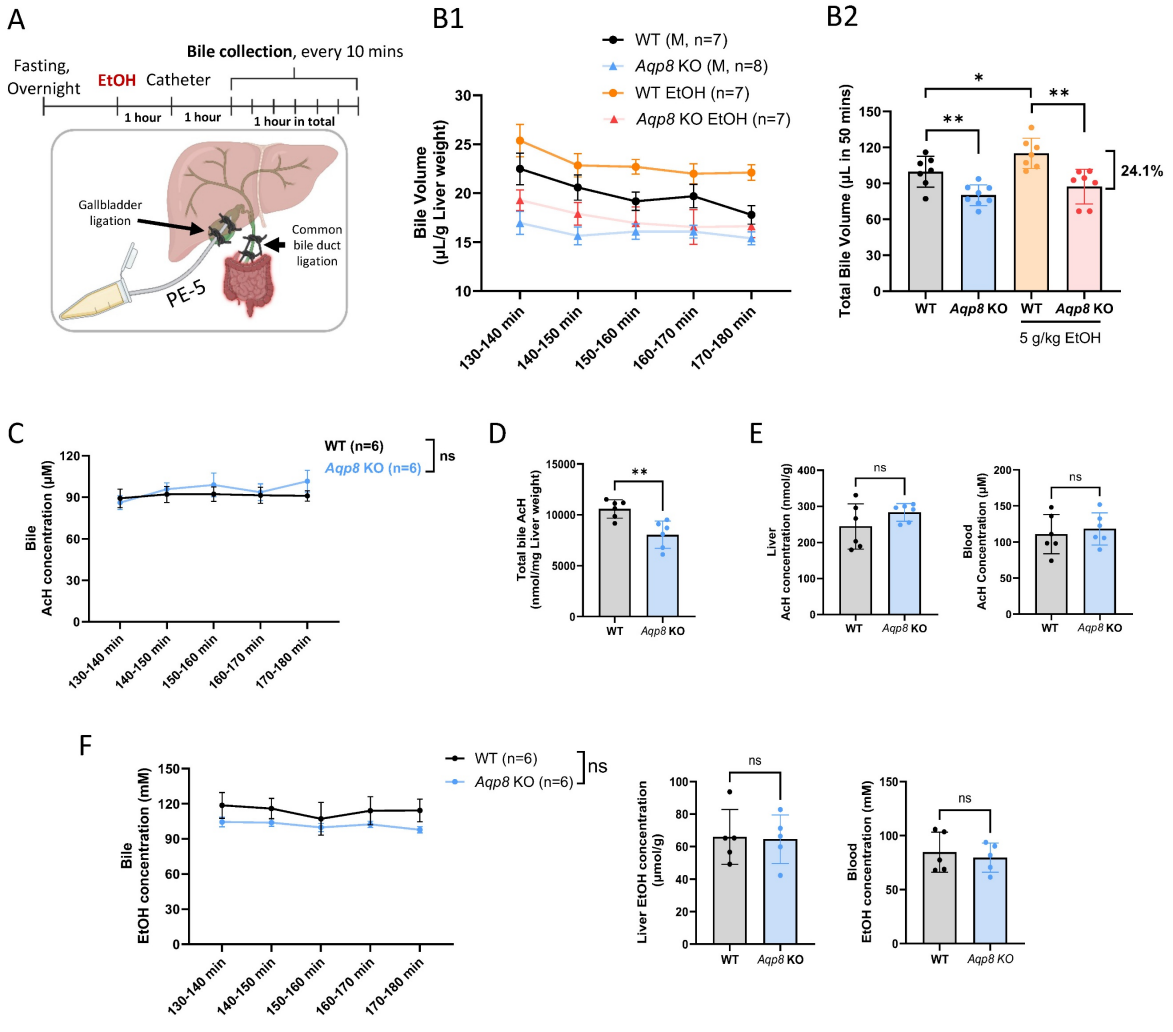
** Alcohol-mediated elevation of bile flow is dependent on AQP8. A.** Working flow of EtOH treatment (5 g/kg) followed by gallbladder cannulation and dynamic bile flow detection (bile collected every 10 mins) in the mouse. The common bile duct was double-ligated, and a PE-5 catheter was placed in the gallbladder under the microscope and fixed with a double-ligation.** B.** 1. Dynamic bile volume and 2. total bile volume (in 50 mins) in WT and *Aqp8* KO mice with or without EtOH gavage in advance (n = 7-8 per group). **C.** AcH concentration in bile in WT and *Aqp8* KO mice between 2 to 3 hours post-EtOH treatment (n = 6 per group). **D.** Total AcH concentration in all collected bile samples (the total concentration was measured by bile volumes multiplied by the concentrations at 5 collecting time points). **E.** Liver and blood AcH concentrations. **F.** Bile, liver, and blood EtOH concentrations. Values represent means ± SD. *P < 0.05, **P < 0.01, ns = no significance. A two-sided Student's t-test was used for the comparison between the two groups.

Our previous study demonstrated that bile efflux plays a key role in mediating AcH distribution in the enterohepatic system, contributing to nearly 30% of AcH clearance in the liver 6. A previous study reported that chronic alcohol consumption increases bile flow in rats 15; however, it remains unclear how acute EtOH administration modulates bile flow. AQP8, localized to the canalicular membrane of hepatocytes, has recently been proven to regulate bile flow 8. Given that bile is more than 95% water 16, we hypothesized that hepatic AQP8 may promote AcH excretion either by directly transporting AcH or by enhancing bile flow. To determine whether binge ethanol exposure enhances bile secretion and to confirm the role of AQP8 in AcH efflux, we monitored dynamic bile flow over 50 minutes, beginning 2-3 hours post-EtOH (5 g/kg) gavage. Briefly, the common bile duct was ligated to block the bile flow. Afterward, a PE-5 catheter was inserted and tightly ligated in the gallbladder under microscopy for exporting the bile, which provided results similar to common bile duct cannulation 8. Mice received EtOH treatment prior to bile duct catheterization. Bile was then collected every 10 minutes for 1 hour, and the initial 10-minute fraction was discarded to exclude residual bile stored in the gallbladder** (Figure 1A, Sup Fig 1A)**.

Across all time points, *Aqp8* KO mice secreted much less bile than their WT counterparts, both under basal and EtOH-treated conditions **(Figure 1B1, B2)**, consistent with the prior literature 8. Intriguingly, EtOH administration significantly increased bile flow in WT mice, but not in *Aqp8* KO mice **(Figure 1B2)**, suggesting that EtOH enhances bile flow in an AQP8-dependent manner. Mechanistically, previous studies have shown that EtOH activates protein kinase A (PKA), which in turn promotes AQP8 translocation to the canalicular membrane through cyclic adenosine monophosphate (cAMP) signaling 17, 18, offering a potential explanation for these observations. Impaired bile flow in *Aqp8* KO mice was further supported by reduced bile volume at each time point **(Sup Fig 1B)** and a 24.1% decrease in total bile volume **(Figure 1B2)**. Although biliary AcH concentrations reached a steady-state and showed no significant difference between groups **(Figure 1C)**, the total amount of AcH excreted via bile was markedly reduced in *Aqp8* KO mice **(Figure 1D)**. This indicates that AQP8 facilitates AcH clearance primarily by promoting bile flow, rather than altering AcH concentration *per se*. Notably, hepatic and blood AcH levels remained comparable between the two groups **(Figure 1E)**, and EtOH levels were also nonsignificant across all samples **(Figure 1F)**, ruling out differences in ethanol metabolism or distribution.

Based on the bile volume (110 µL per gram of liver over 50 minutes) and steady-state biliary AcH concentration (ca. 100 µM), we estimate that more than 10 nmol of AcH was excreted via bile from 1 g liver over 50 mins in WT mice **(Figure 1D)**. This pool of AcH is likely further metabolized in the gut by ALDH2, as part of the enterohepatic detoxification pathway. Collectively, these findings provide direct *in vivo* evidence that AQP8 mediates AcH efflux from the liver to bile, which is enhanced by EtOH-induced, AQP8-dependent bile flow.

### AQP8 facilitates hepatic AcH efflux via bile flow

**Figure 2:**
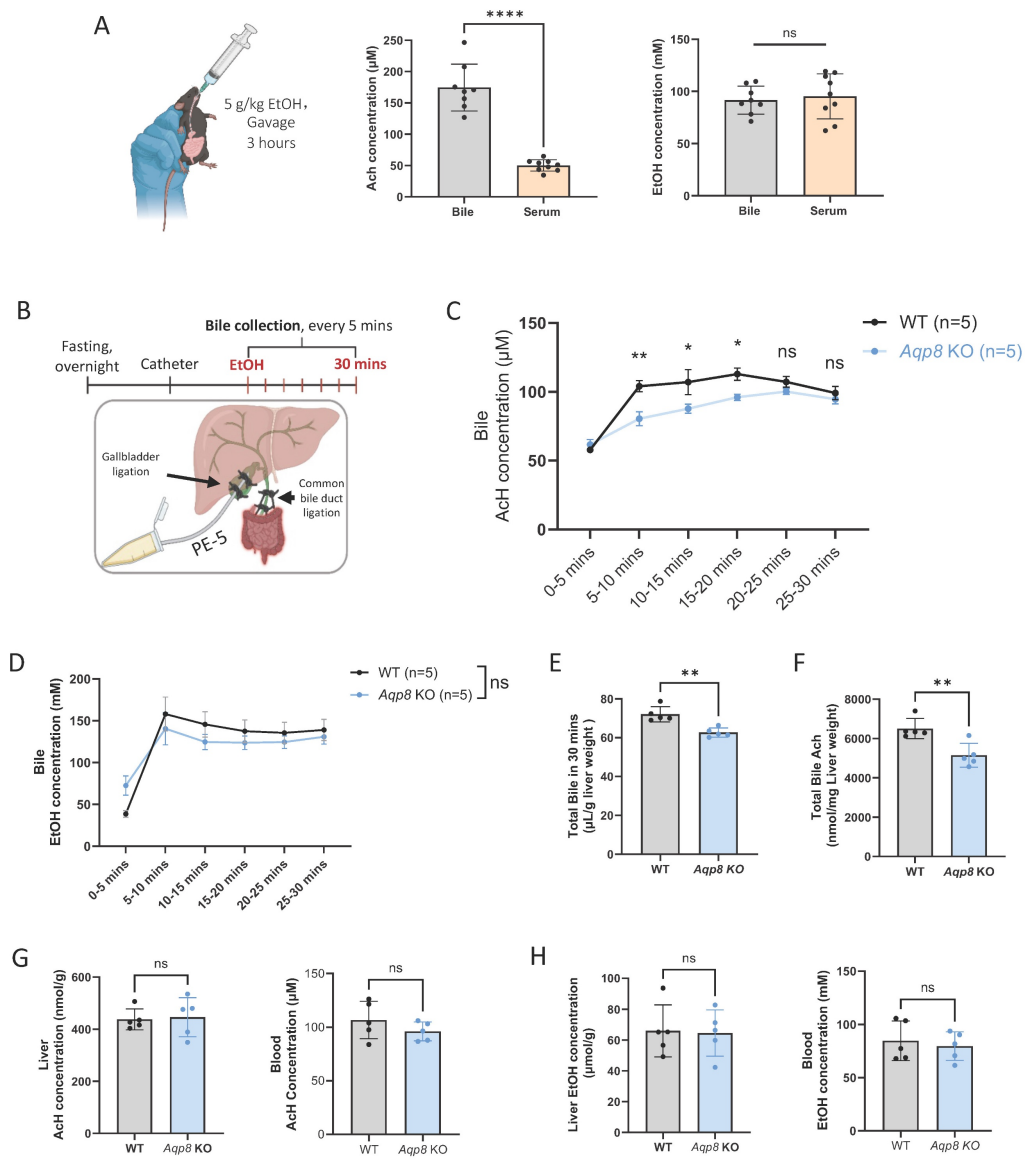
** Hepatic AQP8 directly facilitates AcH efflux via bile flow. A.** Mice were administered with 5g/kg EtOH by gavage. 3 hours post-gavage, bile, serum, and blood AcH and EtOH concentrations were measured by GC-MS (n = 8-9). **B.** Working flow of bile flow surgery followed by EtOH treatment (5 g/kg) and dynamic bile flow detection (bile collected every 5 mins) in the mouse. The common bile duct was double-ligated, and a PE-5 catheter was placed in the gallbladder under the microscope and fixed with a double-ligation.** C.** AcH concentration in bile samples at indicated time points in WT and *Aqp8* KO mice (n = 5 per group). **D.** EtOH concentration in bile samples at indicated time points in WT and Aqp8 KO mice (n=5 per group). **E.** Total bile volume collected in 30 mins. **F.** Total AcH concentrations in all collected bile samples (the total concentration was measured by bile volumes multiplied by the concentrations at 6 collecting time points). **G.** Liver and blood AcH concentrations.** H.** Liver, and blood EtOH concentrations. Values represent means ± SD. *P < 0.05, **P < 0.01, ns = no significance. A two-sided Student's t-test was used for the comparison between the two groups.

Also, we observed that 3 hours after ethanol gavage (5 g/kg), biliary AcH concentrations, not EtOH levels, were over 3-fold higher than those in serum **(Figure 2A)**, highlighting that bile flow is a key route for AcH elimination from the liver. Although we have demonstrated that hepatic AQP8 facilitates AcH efflux through alterations in bile flow, it remains unclear whether AQP8 directly mediates AcH efflux during the early phase following alcohol intake.

To verify this hypothesis, we conducted gallbladder catheterization followed by EtOH gavage and immediately collected bile samples **(Figure 2B)**. Similarly, bile flow surgery was performed as described in Figure 1. Following the surgery, mice were administered 5 g/kg EtOH by gavage, and the bile was collected immediately thereafter. Notably, high AcH levels were detectable as early as 5 mins post-gavage, both in WT and KO mice **(Figure 2C)**, consistent with our earlier findings that bile excretion plays a critical role in AcH clearance 6. More importantly, AcH concentrations were significantly attenuated by *Aqp8* KO in the following 3 collecting time points, supporting that AQP8 directly mediates AcH efflux independently of changes in bile flow **(Figure 2C)**. Of note, the biliary EtOH levels were comparable in the two groups **(Figure 2D)**. Additionally, total bile flow was significantly reduced in *Aqp8* KO mice during the 30-minute collection period **(Figure 2E)**, resulting in decreased total AcH excretion into bile **(Figure 2F),** consistent with the findings in Figure 1D. Despite these changes, liver and blood AcH and EtOH levels remained comparable between WT and KO mice **(Figure 2G, 2H).** Collectively, these results demonstrate that hepatic AQP8 plays a direct role in mediating AcH efflux shortly after alcohol exposure, independent of alterations in bile flow.

### AQP8 serves as a channel facilitating AcH efflux

**Figure 3:**
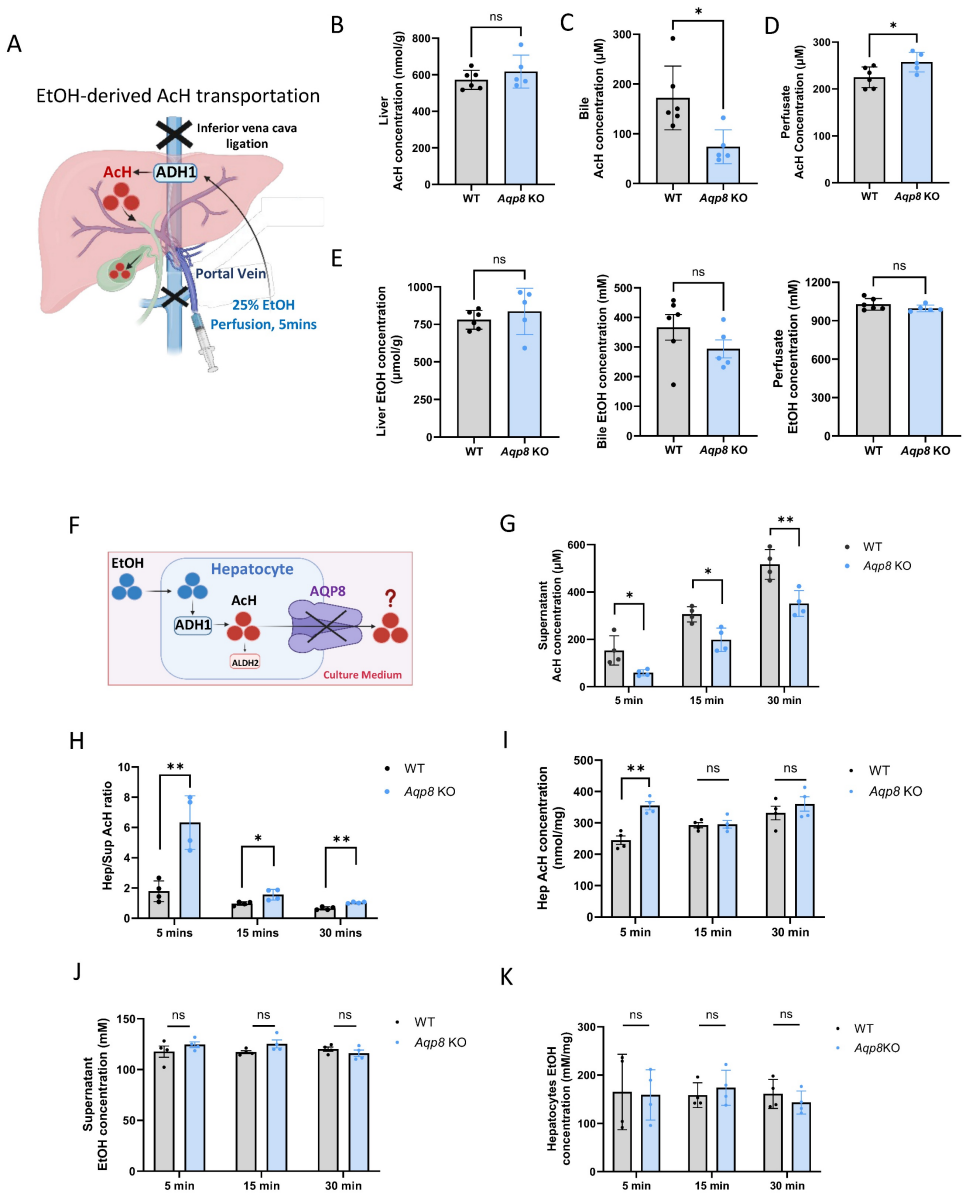
** Hepatic AQP8 is a channel facilitating AcH efflux. A.** Schematically indicating EtOH-derived AcH transport post-EtOH (1 ml in 25%, *vol/vol*, 5 mins) perfusion via the portal vein. **B. C. D.** AcH concentrations in the liver, bile, and perfusate samples in *Aqp8* KO and their WT control mice after EtOH perfusion (n = 5-6 per group). **E.** EtOH concentrations in the liver, bile, and perfusate samples in *Aqp8* KO and their WT control mice after EtOH perfusion (n = 5-6 per group). **F.** The schematic of primary hepatocytes incubated with 100 mM EtOH incubation and AcH excretion mediated by AQP8. **G. H.** Supernatant AcH, hepatocytes/supernatant (Hep/Sup) AcH ratio, and intracellular AcH concentrations at each time point of *Aqp8* KO and WT isolated primary hepatocytes (The experiment was repeated 4 times independently).** J. K.** Supernatant and hepatocytes EtOH concentrations. Values represent means ± SD. *P < 0.05, **P < 0.01, ns = no significance. A two-sided Student's t-test was used for the comparison between the two groups.

To investigate whether AQP8 directly mediates EtOH-derived AcH efflux from the liver, we performed in situ liver perfusion with EtOH in *Aqp8* KO mice and their WT littermates. To prevent systemic circulation, the inferior vena cava was clamped, followed by perfusion of 1 mL of 25% EtOH (*vol/vol*) perfusion for 5 mins to promote endogenous AcH generation within hepatocytes **(Figure 3A)**. EtOH perfusion triggered high levels of hepatic AcH in both WT and KO mice **(Figure 3B)**. Given that portal circulation was blocked in this model, bile flow served as the primary route for AcH excretion. As expected, biliary AcH levels were significantly reduced in *Aqp8* KO mice compared to WT controls **(Figure 3C)**, supporting the role of AQP8 in mediating AcH efflux via bile. Moreover, elevated AcH concentrations in the perfusion solution effluent from *Aqp8* KO livers **(Figure 3D)** may reflect compensatory efflux across the sinusoidal membrane due to impaired canalicular AcH transport. EtOH concentrations remained comparable between groups, ruling out differences in ethanol metabolism **(Figure 3E)**. To mimic EtOH-derived AcH secretion *in vitro*, we isolated primary hepatocytes from *Aqp8* KO and WT mice and immediately incubated them in suspension with 100 mM EtOH in cell culture medium (**Figure 3F**). After 5, 15, and 30 mins of incubation, AcH levels were separately detected and measured in both the hepatocytes and the culture medium. Intriguingly, AcH levels in the supernatant were significantly lower in *Aqp8* KO cells at all three time points **(Figure 3G)**, accompanied by a higher intracellular-to-supernatant AcH (Hep/Sup) ratio **(Figure 3H)**, indicating impaired AcH efflux in the absence of AQP8. Additionally, elevated AcH level in the *Aqp8* KO hepatocytes at the early time point (5 mins) may be due to the impaired AcH excretion, leading to intracellular AcH accumulation **(Figure 3I)**. Supernatant and hepatocyte EtOH levels showed no differences between the two groups **(Figure 3J, 3K)**. Similar defects were observed when hepatocytes were treated directly with exogenous AcH **(Sup Fig 2A)**.

Collectively, these findings provide strong evidence that hepatic AQP8 functions as a channel, directly facilitating the excretion of liver-generated AcH.

### AQP8 enhances alcohol consumption

**Figure 4:**
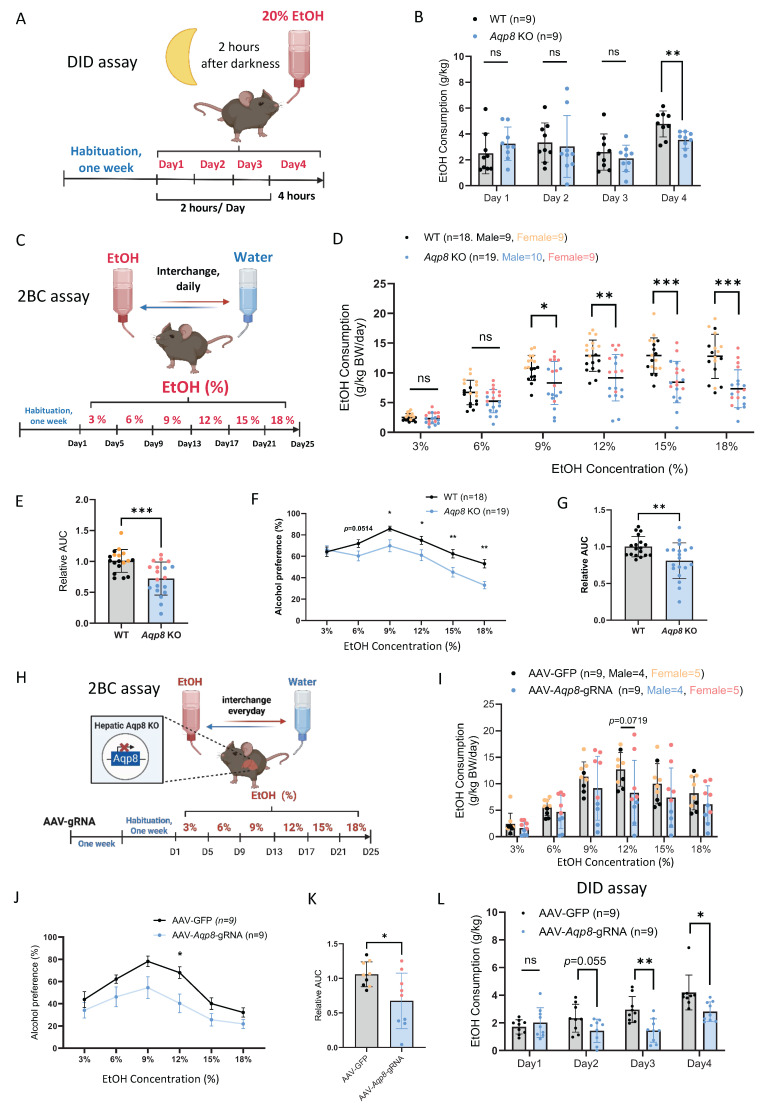
** AQP8 enhances alcohol consumption. A.** Schematic elucidating a Drinking in the Dark (DID) paradigm. Mice were single-housed and acclimated for one week. Three hours after the lights were turned off (darkness) in the animal housing room, the water bottle was replaced with an ethanol-containing bottle (20% vol/vol) for 2 hours on days 1-3 and 4 hours on day 4. The amount of ethanol consumed was weighed and recorded daily.** B.** Daily EtOH consumption of DID in WT and *Aqp8* KO mice (n=9 per group). **C.** Schematic elucidating a 2-Bottle Choice (2BC) paradigm. Mice were acclimated for one week and single-housed with free access to a water bottle or an escalating concentration of EtOH bottle (3/6/9/12/15/18%). The position of the two bottles was switched daily to diminish positional preference. EtOH and water consumption were measured every 4 days. **D.** EtOH consumption in WT and *Aqp8* KO male and female mice at each percentage (n = 9-10 per sex per group). **E.** Statistics of the relative AUC of EtOH consumption. **F. G.** Alcohol preference curves at each percentage and statistics of the relative AUC in WT and *Aqp8* male and female mice (n = 9-10 per sex per group). **H.** Schematic elucidating AAV-gRNA induced liver-specific *Aqp8* KO followed by 2BC paradigm. The 2BC paradigm was performed with the same protocol. **I.** EtOH consumption in control (AAV-GFP) and hepatic *Aqp8* KO (AAV*-Aqp8*-gRNA) mice at each percentage (n = 9 per group). **J. K.** Alcohol preference curves at each percentage and statistics of the relative AUC in control (AAV-GFP) and hepatic Aqp8 KO (AAV*-Aqp8*-gRNA) mice (n = 9 per group). **L.** Daily EtOH consumption of DID in control (AAV-GFP) and hepatic *Aqp8* KO (AAV*-Aqp8*-gRNA) mice (n = 9 per group). Values represent means ± SD or SEM. *P<0.05, **P < 0.01, ***P < 0.001, ns = no significance. A two-sided Student's t-test was used for the comparison between the two groups.

Our previous publications have shown that altered peripheral AcH distribution and clearance can influence alcohol drinking behavior 6. To assess the role of AQP8-mediated AcH secretion in modulating drinking behavior, we conducted a drinking-in-the-dark (DID) assay to measure binge-like consumption of 20% alcohol **(Figure 4A)** 19. As shown in **Figure 4B**, *Aqp8* KO mice consumed significantly less alcohol on day 4, indicating that AQP8 promotes high-concentration binge alcohol intake. We next employed a two-bottle choice (2-BC) paradigm to evaluate voluntary ethanol consumption and preference in both male and female WT and *Aqp8* KO mice **(Figure 4C)**. Compared with WT mice, *Aqp8* KO mice exhibited significantly less ethanol consumption starting at 9% concentration **(Figure 4D)** and showed a marked reduction in total ethanol intake **(Figure 4E)**. Furthermore, ethanol preference was significantly lower in *Aqp8* KO mice, particularly at higher ethanol concentrations (15% and 18%) **(Figure 4F, 4G)**. These differences were consistently observed in both sexes **(Sup Fig 3A)**.

To control the potential confounding effects of taste sensitivity, we performed a saccharin two-bottle choice test using 0.01% and 0.2% saccharin solutions **(Sup Fig 3B)** 20. Both WT and *Aqp8* KO mice demonstrated comparable saccharin consumption and strong preference, indicating that the suppressed ethanol drinking in *Aqp8* KO mice is not attributable to altered taste preference. Moreover, AcH clearance was significantly delayed in the liver, bile, and blood samples post 6 to 9 hours of alcohol gavage in *Aqp8* KO mice, suggesting AQP8 is important to speed up the liver and systemic AcH clearance **(Sup Fig 3C, 3D)**. The delayed AcH clearance might contribute to the suppressed drinking behavior in KO mice.

To determine whether hepatic AQP8 specifically governs alcohol consumption, we selectively depleted liver AQP8 expression using AAV-delivered guide RNA (gRNA) in Cas9-expressing mice. One week after the intravenous AAV-*Aqp8*-gRNA injection, mice were tested using the 2-BC paradigm **(Figure 4H)**. Consistent with the genetic global KO model, hepatic-specific *Aqp8* KO (AAV-*Aqp8*-gRNA) mice exhibited a clearly suppressed alcohol consumption pattern, with a significantly lower drinking preference at 12% EtOH concentration **(Figure 4I, 4J)** and an overall lower drinking compared with controls **(Figure 4K)**. Moreover, hepatic *Aqp8* KO dramatically reduced alcohol consumption in the DID assay **(Figure 4L)**, reinforcing the conclusion that hepatic AQP8 plays a critical role in promoting high-concentration alcohol intake.

### Alcohol-induced hepatic Aqp8 deficiency aggravates ALD

**Figure 5:**
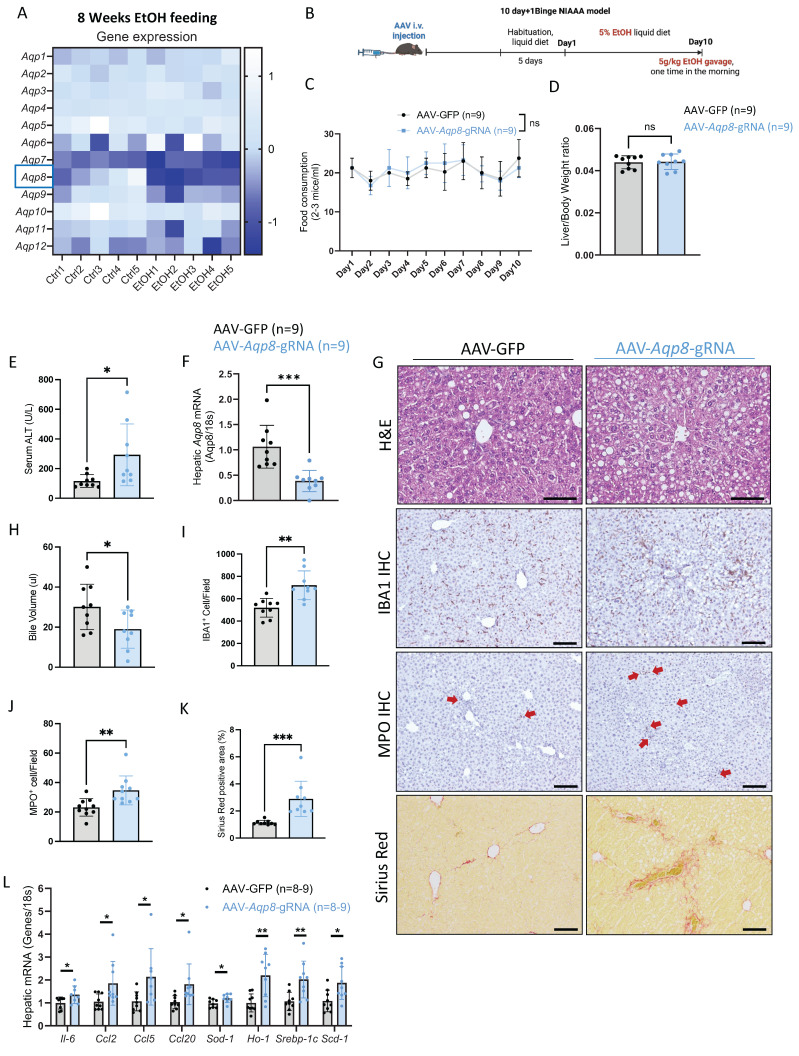
** Alcohol-induced hepatic AQP8 deficiency aggravates ALD. A.** Twelve isoforms of hepatic *Aqps* gene expression changes in 8-week 3-binge ALD model (n = 5). **B.** Working flow of hepatic *Aqp8* knockout followed by the NIAAA model. **C.** Food consumption in control (AAV-GFP) and hepatic *Aqp8* knockout (AAV*-Aqp8*-gRNA) mice (n = 9). **D.** Liver-to-Body weight ratio in the two indicated groups (n = 9). **E.** Serum ALT levels in the two indicated groups (n = 9). **F.** Hepatic *Aqp8* mRNA expression in the two indicated groups (n = 9). **G.** Representative liver H&E staining, IBA1, MPO IHC staining, and liver Sirius red staining in the two indicated groups. **H.** Bile volume analysis (n = 9). **I. J.** IBA and MPO positive cell counts (n = 9). **K.** Positive Sirius Red staining area analysis (%), (n = 9). **L.** Hepatic proinflammatory genes (*Il-6, Ccl2, Ccl5, Ccl20*), oxidative stress genes (*Sod-1, Ho-1*), and lipogenesis-related genes (*Srebp-1c, Scd-1*) mRNA expression (n = 8-9).

Impaired bile flow is a key pathogenic mechanism in cholestasis, a condition commonly observed in alcohol-associated hepatitis that contributes to hepatocellular injury and liver inflammation which can ultimately progress to fibrosis and cirrhosis 21-23. Notably, in our 8-week chronic ethanol plus 3-binge ALD model, liver *Aqp8* is the most significantly downregulated aquaporin after ethanol administration, as shown by the RNA-seq data **(Figure 5A)**. To determine whether hepatic AQP8 deficiency contributes to ALD pathogenesis, we subjected the hepatic-specific *Aqp8*-knockout mice (AAV*-Aqp8*-gRNA) to the NIAAA alcohol feeding model **(Figure 5B)** 24. No significant differences were observed in EtOH diet consumption or liver-to-body weight ratio between the control (AAV-GFP) and the AAV*-Aqp8*-gRNA groups **(Figure 5C, 5D)**. However, AAV*-Aqp8*-gRNA mice exhibited significantly elevated serum ALT levels, indicating exacerbated liver injury **(Figure 5E)**. Quantitative PCR (qPCR) confirmed that hepatic *Aqp8* mRNA expression was reduced by approximately 60% following AAV-guided RNA delivery, which was associated with a significant reduction in bile volume collected from the gallbladder **(Figure 5F-5H)**.

Histologically, AAV*-Aqp8*-gRNA livers showed more extensive lipid accumulation and hepatocyte ballooning, particularly in the pericentral area (zone 3), as demonstrated by H&E staining **(Figure 5G)**. Immunohistochemical (IHC) staining revealed increased infiltration of ionized calcium-binding adaptor molecule 1 (IBA1) positive macrophages and myeloperoxidase (MPO) positive neutrophils in AAV*-Aqp8*-gRNA livers, indicating heightened hepatic inflammation **(Figure 5G, 5I, 5J)**. Furthermore, the positive Sirius Red staining area was significantly increased in AAV*-Aqp8*-gRNA livers, indicating progressive liver fibrosis **(Figure 5G, 5K)**. Consistent with these histological findings, mRNA levels of proinflammatory genes (*Il6, Ccl2, Ccl5, Ccl20*), lipogenesis-related genes (*Srebp-1c, Scd-1*), and oxidative stress markers (*Sod1, Ho1*) were significantly upregulated in AAV*-Aqp8*-gRNA mice **(Figure 5L)**. Collectively, these findings demonstrate that chronic alcohol consumption impairs hepatic AQP8, which highly contributes to impaired bile flow and exacerbates liver inflammation, steatosis, and oxidative stress in the context of ALD.

### Hepatic AQP8 overexpression alleviates ALD progression

Given AQP8 deficiency contributes ALD pathogenesis and the known roles of hepatic AQP8 in AcH distribution and bile secretion, we hypothesized that restoring AQP8 expression would enhance AcH excretion via bile flow, thereby reducing hepatic AcH accumulation and mitigating alcohol-induced liver injury.

**Figure 6:**
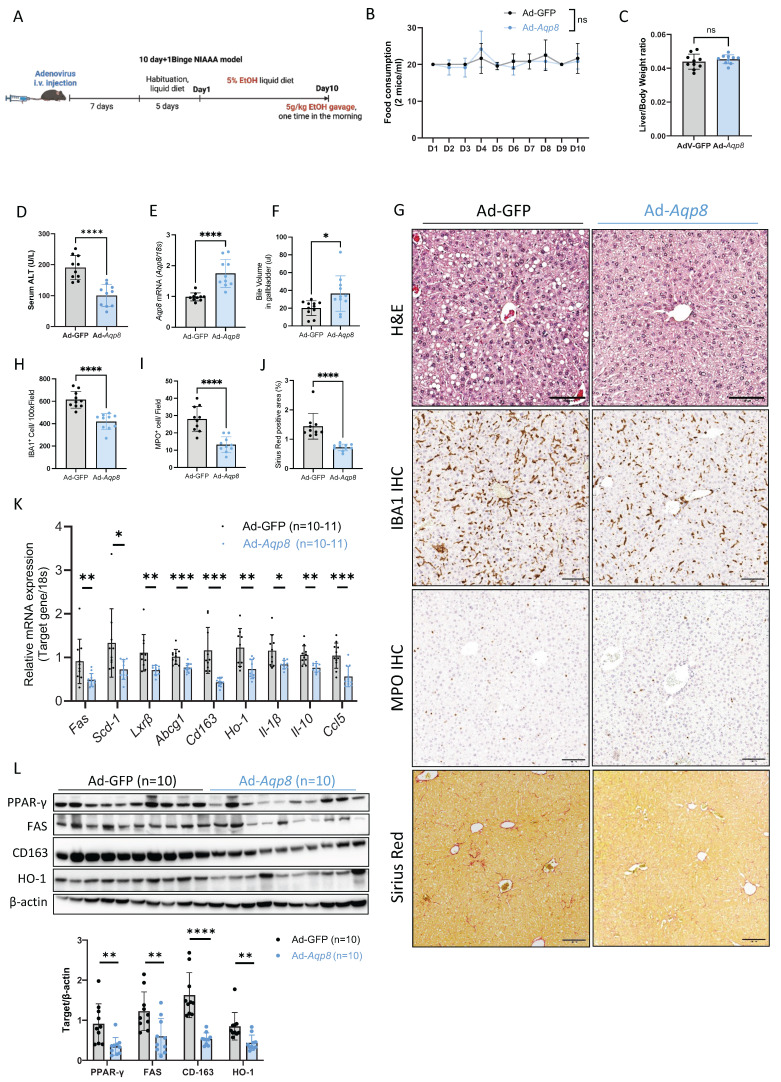
**Hepatic AQP8 overexpression alleviates ALD. A.** Working flow of adenovirus-mediated hepatic *Aqp8* overexpression followed by the NIAAA model. **B.** Food consumption in adenovirus control (Ad-GFP) and adenovirus-mediated hepatic *Aqp8* overexpression (Ad-*Aqp8*) mice (n = 10-11). **C.** Liver-to-Body weight ratio in the two indicated groups (n = 10-11). **D.** Serum ALT levels in the two indicated groups (n = 10-11). **E.** Hepatic *Aqp8* mRNA expression in the two indicated groups (n = 10-11). **F.** Bile volume analysis (n = 11). **G.** Representative liver H&E staining, IBA1, and MPO IHC staining, and Sirius Red staining in the two indicated groups. **H. I.** IBA and MPO positive cell counts (n = 10). **J.** Positive Sirius Red staining area analysis (%), (n = 9). **K.** Hepatic lipid metabolism and transporter gene (*Fas, Scd-1, Lxr-β, Abcg1*) and (anti)-inflammatory gene (*Cd163, Ho-1, Il-1β, Il10, Ccl5*) mRNA expression (n = 10-11). **L.** Hepatic PPAR-γ, FAS, CD163, and HO-1 protein expression and their densitometric analysis (corrected by β-actin, n = 10 per group).

To test this, we overexpressed *Aqp8* in the liver using an adenovirus vector driven by an albumin promoter, followed by ALD induction using the NIAAA model **(Figure 6A)**. No significant differences were observed in EtOH diet consumption or liver-to-body weight ratio between the control adenovirus (Ad-GFP) and the *Aqp8*-overexpression (Ad-*Aqp8*) groups **(Figure 6B, 6C)**. Importantly, serum ALT levels were significantly reduced in Ad-*Aqp8* mice **(Figure 6D)**, indicating amelioration of liver injury. *Aqp8* overexpression significantly increased hepatic *Aqp8* mRNA and bile volume collected from the gallbladder **(Figure 6E-6G)**, supporting the role of AQP8 in enhancing bile flow and alleviating early-stage ALD. Histologically, H&E staining revealed fewer lipid droplets in zone 3 **(Figure 6G)**. Furthermore, IHC showed reduced IBA1 and MPO staining in the liver, indicating decreased infiltration of macrophages and neutrophils, respectively **(Figure 6G, 6H, 6I)**. The Sirius Red staining indicated liver fibrosis was also decreased in Ad-*Aqp8* mice **(Figure 6G, 6J)**.

In addition, in the livers of Ad-*Aqp8* mice, the expression of genes involved in lipid metabolism and cholesterol transport, including *Fas, Scd-1, Lxrβ, and Abcg1*, as well as PPAR-γ and FAS protein levels **(Figure 6J, 6K)**, were significantly decreased, consistent with improved hepatic lipid metabolism. Additionally, both pro-inflammatory and anti-inflammatory genes, such as *Cd163, Ho-1, Il-1β, Il-10, and Ccl5*, along with CD163 and HO-1 protein expression **(Figure 6K, 6L)**, were markedly suppressed, supporting the notion of alleviated liver inflammation.

In contrast to liver-specific *Aqp8* KO, global *Aqp8* KO unexpectedly ameliorated ALD, as evidenced by significantly reduced serum ALT levels and liver-to-body weight ratio in both sexes **(Sup Fig. 4A, 4B)**, despite comparable food consumption among groups **(Sup Fig. 4C)**. Consistent with the RNA-seq data shown in Figure 5A, the 5% EtOH diet markedly suppressed hepatic *Aqp8* mRNA expression **(Sup Fig. 4D)**. Reduced lipid deposition in the pericentral (Zone 3) region indicated improved lipogenesis in KO mice **(Sup Fig. 4E)**. In addition, staining for MPO⁺ neutrophils and IBA1⁺ macrophages revealed significantly decreased inflammatory infiltration in KO livers, further indicating ameliorated hepatic inflammation **(Sup Fig. 4F)**. These seemingly contradictory findings between liver-specific versus global *Aqp8* KO suggest that AQP8 in extrahepatic tissues contributes substantially to ALD progression.

To further clarify the role of hepatic AQP8 in ALD independent of extrahepatic AQP8, we overexpressed *Aqp8* in the liver on a global KO background (*Aqp8* KO ^Ad-*Aqp8*^). The food consumption remained comparable between groups **(Sup Fig. 5A)**. Compared with GFP littermate controls (*Aqp8* KO ^Ad-GFP^), *Aqp8* KO ^Ad-*Aqp8*^ mice exhibited significantly lower serum ALT, indicating reduced liver injury **(Sup Fig. 5B)**. Notably, ALT levels in *Aqp8* KO ^Ad-GFP^ mice were comparable to those in the global KO mice (~100 U/L; **Sup Fig. 5A and 4A**), supporting that hepatic *Aqp8* overexpression improved ALD independently of global AQP8 status. The efficiency of hepatic Aqp8 overexpression was confirmed by elevated mRNA expression and increased bile volume **(Sup Fig. 5C, 5D)**.

Taken together, these findings demonstrate that hepatic *Aqp8* overexpression enhances bile flow, facilitates AcH excretion, and contributes to the improvement of lipid metabolism and reduction of liver inflammation in ALD.

## Discussion

**Figure 7:**
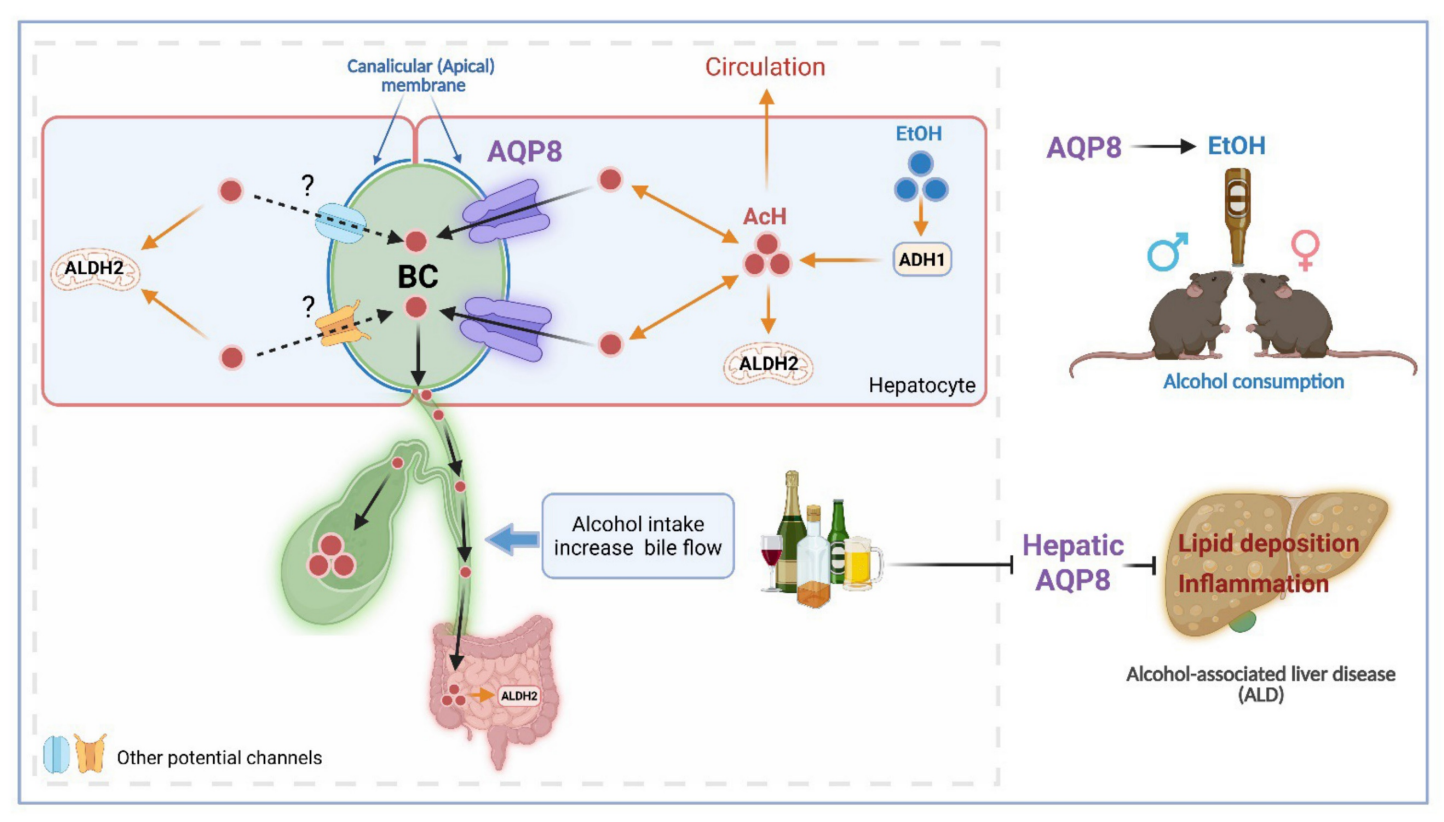
** Hepatic AQP8 promotes alcohol consumption and protects against alcohol-induced liver injury by facilitating acetaldehyde excretion.** Hepatic AQP8 is localized to the canalicular membrane of hepatocytes, where it facilitates AcH efflux into bile. This AQP8-mediated AcH excretion enhances overall AcH clearance along the liver-gut axis, thereby accelerating hepatic detoxification. Efficient AcH clearance decreases peripheral AcH accumulation, which may reduce aversive responses and promote voluntary alcohol consumption in both male and female mice. Consequently, enhanced AQP8-dependent AcH excretion ameliorates alcohol-induced liver injury in ALD. AQP8, aquaporin 8, AcH, acetaldehyde, EtOH, ethanol, ADH1, alcohol dehydrogenase 1, ALDH2, aldehyde dehydrogenase 2, BC, bile canaliculi.

The current study is the first to identify hepatic AQP8 as a channel protein that alters AcH distribution by facilitating hepatic AcH excretion into bile. This function appears to influence ALD progression and increase alcohol consumption, likely due to accelerated AcH clearance and enhanced bile flow **(Figure 7)**.

Previous studies found that chronic alcohol feeding increased bile flow 15, 25, and a recent study reported that AQP8 mediated bile flow and further affected gallstone formation 8. Collectively, these studies strongly support our findings that binge drinking increases bile flow, which is regulated by AQP8. Notably, AQP8-mediated AcH excretion is more obvious at early phases after EtOH treatment, and lower biliary AcH concentrations at early time points (5-20 mins) turn into a steady-state level at later (2-3 hours) post-EtOH gavage. These findings highlight that hepatic AQP8 mediates AcH excretion directly via bile and indirectly via enhanced bile flow.

Our prior work demonstrated that simultaneous deletion of the *Aldh2* gene in both the liver and gut synergistically reduces alcohol consumption, with bile flow plays a key role in regulating AcH clearance along the liver-gut axis 6. In line with this, we observed that biliary AcH becomes detectable within 5 minutes of ethanol exposure, reaching concentrations as high as 50 µM, supporting our earlier conclusion that bile flow serves as a critical AcH clearance route. In our current study, dynamic bile collection from 5 minutes up to 3 hours post-gavage, along with measurements at 6 hours, revealed a consistently high biliary AcH concentration (70-100 µM). Approximately 120 µL of bile is secreted per hour per gram of liver in mice. By comparison, the human liver secretes 0.7 to 1 L of bile daily, at a rate of 30-40 mL/hour 26, suggesting that during the 3 hours following binge drinking, 90-120 mL of bile with high AcH content could be excreted in humans. This implies that changes in bile flow, whether impaired or enhanced, can substantially influence liver AcH excretion and hepatic and intestinal AcH metabolism.

In the last decades, the effects of AcH on the regulation of drinking behavior have been under debate. It is becoming clear that AcH regulates drinking behavior depending on the localization of its accumulation. A large amount of data suggested that AcH contributes to or even mediates the reinforcing effects of alcohol consumption, especially escalating AcH levels in some regions in the brain 27, 28. However, the high peripheral AcH levels dramatically suppressed the drinking preference, for instance, AcH accumulation due to ALDH2 inactivity 29. In our recent study, ALDH2 expressed in the liver-gut axis largely contributes to the systemic AcH clearance. ALDH2 deficiency in the liver and gut synergistically suppressed voluntary alcohol consumption due to the high peripheral AcH level and aversive effects 6. Consistent with our previous findings that dynamic bile flow regulates alcohol drinking behavior 6, *Aqp8* KO mice showed reduced ethanol preference, largely due to impaired bile secretion. Notably, liver-specific *Aqp8* KO significantly suppressed high-concentration (20%) ethanol intake and reduced drinking preference, highlighting a key role for hepatic AQP8 in promoting AcH efflux and facilitating alcohol consumption. In addition, the delayed clearance of AcH from the liver and peripheral blood observed in *Aqp8* KO mice further supports the notion that AQP8-facilitated AcH excretion contributes to systemic AcH clearance and, consequently, influences alcohol consumption. However, the effects of AQP8 from other organs on drinking behavior, for instance, the intestine or the brain, are not excluded. These are also critical organ for metabolizing AcH and controlling drinking behavior 9. Specific *Aqp8* KO in the intestine or brain is needed to identify the role of AQP8-facilitated AcH transport in regulating drinking behavior. In general, targeting AcH elimination, through both its metabolism and transport along the liver-gut axis via bile flow, may represent a novel therapeutic approach for the treatment of AUD. Given that at least 12 other AQP isoforms are expressed throughout the body 12, it is plausible that additional AQPs may also participate in AcH transport. Therefore, the broader role of AQPs-mediated AcH clearance and their collective impact on alcohol-related behaviors warrant systematic investigation.

Finally, hepatic *Aqp8* expression was markedly suppressed in chronic alcohol feeding models, suggesting impaired bile flow, which may contribute to cholestasis and promote liver injury. Consistent with this, liver-specific *Aqp8* deficiency aggravated ALD pathogenesis in our knockout mouse model, as evidenced by reduced bile secretion, increased hepatic inflammation, and lipid accumulation. Conversely, restoration of hepatic *Aqp8* expression significantly enhanced bile flow, improved lipid metabolism, and attenuated hepatic inflammation in the NIAAA model. These findings support a novel role for AQP8 in facilitating bile-mediated AcH excretion and protecting against ALD. Targeting the AQP8-mediated AcH clearance and bile flow pathway may represent a promising therapeutic strategy for ALD. In addition, chronic alcohol significantly induced hypoxia and hypoxia-inducible factor 1α (HIF-1α) level in the liver, which contributes to liver inflammation and steatosis in ALD 30, 31. Given that HIF-1α has been reported as a transcriptional regulator suppressing *Aqp8* 8, chronic alcohol intake may suppress *Aqp8* levels by activating HIF-1α. Nonetheless, further mechanistic studies are warranted to fully elucidate the regulatory networks and downstream effects of AQP8 function in the liver under chronic ethanol exposure.

In summary, by using multiple *in vivo* and *in vitro* models combined with targeted surgical approaches, the current study provides fundamental insights into the mechanisms of AcH transport. Hepatic AQP8 is identified as a key modulator of AcH distribution that facilitates hepatic AcH efflux both directly, through biliary excretion, and indirectly, by enhancing bile flow. This AQP8-mediated pathway plays a crucial role in regulating AcH clearance within the enterohepatic circulation. Furthermore, hepatic AQP8 modulates alcohol consumption and influences the progression of ALD, offering new perspectives for the clinical management of both AUD and ALD.

## Methods

### Mouse experiments

*Aqp8* KO mice on the C57BL/6J background were purchased from Cyagan Biosciences, Cas9 mice (Jax #:026179) on the C57BL/6J background were purchased from the Jackson Laboratory. All animal experiments were approved by the National Institute on Alcohol Abuse and Alcoholism (NIAAA) Animal Care and Use Committee. Animals were housed under a 12-h light/dark cycle with room temperature of 22 ºC ± 1 ºC and humidity of 30-70%. Mice had free access to food and water unless otherwise specified. Mice between 6 and 24 weeks old were used for all experiments. Sex was not considered as a special factor in the study design. *Aqp8*-Adenovirus (Ad-*Aqp8*) with the Albumin promoter was purchased from Signagen Laboratories to mediate Aqp8 overexpression in the liver.

### Generation of liver-specific *Aqp8* KO mice

sgRNA sequences (GTGTAGTATGGACCTACCTG and TAATGAGCAGTCCTACAAAG) targeting *Aqp8* gene were cloned into AAV sgRNA expression plasmid (Addgene #174540), and then packaged into AAV8 particles (OriGene Technologies), and injected to Cas9 mice at the 10^11 GC/mouse to generate hepatic *Aqp8* KO mice (AAV-*Aqp8*-gRNA).

**Liver ethanol perfusion:** WT and *Aqp8* KO were anesthetized by inhalation of 2% isoflurane during the liver perfusion. As we described before 6, the liver and portal vessels were well exposed by surgery. The inferior vena cava and hepatic artery were carefully isolated and ligated with a 4-0 silk suture (Cat. #: ETHVCP392H) and positioned another 4-0 silk suture under the arch of the portal vein proximal to the liver. The second silk suture was placed distal to the inferior mesenteric vein, further downstream from the liver. Once the sutures are in place, cannulate the portal vein with a 22-G catheter (Jelco® IV Catheters). Tie the first suture past the catheter tapper. And secure the lower portion of the catheter with the second suture. When the catheter is secured, insert a 3 mL syringe long into the catheter to inject 1 mL ethanol (25% *vol/vol*) into the liver. 5 minutes later, the bile was taken from the gallbladder, and perfusion liquid was collected from the catheter for AcH measurement.

**Acute ethanol gavage:** Mice received an oral gavage of 5 g/kg ethanol with a 20G gavage needle (Cat. #: AFN2038C). Mice were placed on a heating pad at 38°C throughout the experiment to prevent hypothermia. After the indicated time points, samples were collected.

**Dynamic bile flow detection**: WT and *Aqp8* KO mice were anesthetized by inhalation of isoflurane with 3% for induction and 2% for maintenance during the surgery. The heating pad will be placed under the mouse to keep the body temperature. The mouse will be placed in the supine position, and a 2cm wide incision starting from the xiphoid process will be made along the linea alba of the mouse. The liver and gallbladder will be exposed under the surgical scope. The lower part of the common bile duct, which connects to the duodenum, will be carefully isolated and ligated with a 4-0 silk suture (Cat. #: ETHVCP392H), and another 4-0 silk suture will be positioned around the gallbladder. Once the sutures are in place, a cannulate will be placed into the gallbladder with a PE-5 polyethylene catheter (Jelco® IV Catheters) 8, 32, 33. Once the catheter cannulates into the gallbladder, it will be ligated with a suture. The bile from the gallbladder will be collected via the catheter. The collected volume will reflect the bile flow in the following 10 minutes to 1 hour. After finishing the bile collection, mice will be euthanized by cervical dislocation.

For the detection of the bile flow rate with alcohol drinking, mice will be gavaged with 5g/kg EtOH (31.25% *vol/vol*) before or after the gallbladder cannulation, and the bile flow will be measured at various time points post-ethanol gavage. Bile, liver, and blood samples will be harvested for EtOH and AcH measurement.

**Primary hepatocyte isolation and EtOH/AcH incubation:** WT and *Aqp8* KO were anesthetized by inhalation of 2% isoflurane during the liver perfusion. The liver and portal vessels were well-exposed during the surgery. A 4-0 silk suture under the arch of the portal vein proximal to the liver. Once the suture is in place, cannulate the portal vein with a 22-G catheter (Jelco® IV Catheters). Tie the suture past the catheter tapper and cut out the inferior vena cava. Perfuse the liver with 1x EGTA solution (50 ml) for 5 min at 37 ℃ at a flow rate of 4 ml/min and then via recirculation with collagenase (50 ml) until the hepatic parenchyma beneath the capsule appeared liquefied (important). During the perfusion, it is necessary to press the inferior vena cava with an iris-curved forcep 30-40 sec for 2-3 times to keep enough flow pressure in the liver. Remove the liver and place the liver in a sterile Petri dish (60 x 15mm), add digestion buffer (approx. 3 ml), and cut the liver with iris scissors (mince into ~1mm fragments). Transfer liver fragments to a 50 ml tube with a total of 20 ml digestion buffer and seal the cap. Shake for 20 ~ 30 min at 100 rpm in a 37 ℃ incubator. The fragments of the liver were cooled down on ice and filtered with 70 µm nylon (50ml tube). Add GBSS buffer up to 50ml and centrifuge (400 rpm, 5 min, 3 times at room temperature) to remove dead cells, the deposit is Hepatocytes. After isolation, isolated cells were counted under the microscope. Cells were aliquot (5*10^5) into 0.5 ml medium and further incubated with 200 mM EtOH/ 2 mM AcH (37 ℃, 5mins, in the Thermomixer) (ALDH2 inhibitor added) 5/15/30 mins, after incubation, cells were centrifuged (4 ℃, 800 RPM, 5 mins). The supernatant and cell pellet were separately collected for the GC/MS detection, all cellular AcH/ EtOH concentrations measured by GC/MS were corrected by protein concentrations detected by BCA.

**Mouse model of chronic ethanol diet feeding:** For the chronic ethanol-induced liver injury assessment, the NIAAA mouse model developed by our lab was used in the current study 24. Generally, ethanol-fed or pair-fed mice were initially fed with the control Lieber-DeCarli diet (Bio-Serv, Lot#289767.00) ad libitum for 5 days to acclimatize them to a liquid diet and tube feeding. Then, the mice of the ethanol group (EtOH group) were fed with ethanol Lieber-DeCarli diet (Bio-Serv, Lot#265380.00) containing 5% (vol/vol) ethanol for 10d and the control groups were pair-fed with the isocaloric control diet. On Day 11, ethanol-fed and pair-fed mice were administered oral gavage with a single dose of ethanol (5 g/kg) or isocaloric maltose dextrin solution, respectively. The mice were euthanized 9 hours later, and tissues were collected for further analysis.

**Drinking in the dark (DID) assay:** As we described before 6, to avoid disturbance on the behavioral test, the DID procedure was performed in a quiet, dedicated housing room on a standard 12-h light-dark cycle. Mice were single-housed and acclimated for one week with free access to a standard chow diet and drinking water. During the 4-day assay, 3 hours after lights were turned off (into darkness) in the animal housing room, the water bottle was replaced with an ethanol-containing bottle (20% *vol/vol*) for 2 hours (days 1-3) and 4 hours on day 4. Bottle replacement was performed while a red lamp was temporarily turned on to minimize the disruption of circadian rhythms. The amount of ethanol consumed was weighed and recorded daily.

**Alcohol two-bottle choice (2BC) assay:** 6-8 weeks-old male and female mice in the indicated groups were single-housed for 1 week before the 2BC assay. After one week of habituation, mice were given the free choice between 2 bottles, one of them contained normal drinking water while the other one contained escalating alcohol solutions prepared with drinking water (typically 3%, 6%, 9%, 12%, 15%, and 18% *vol/vol*). Alcohol and water drinking volumes were measured every two days, and bottle positions were interchanged daily to prevent learned preference 34, 35. The body weight of all mice was measured after every two concentration periods.

**Sweetness two-bottle choice (2BC) test:** Similar to alcohol 2BC, mice got free access to water bottles or saccharine (sweet taste) bottles at 0.01% and 0.2% (weight/vol) 20. During the assay, bottle positions were interchanged daily to prevent learned preference. The mice received each concentration for 4 days, and the consumption of two bottles was measured every 2 days. Body weight was measured before and after the assay.

### Ethanol and acetaldehyde measurements by gas chromatography/mass spectrometry (GC/MS) Positive Chemical Ionization

Tissues were collected and frozen over dry ice or liquid nitrogen and stored at - 80 °C until further analysis. A high-throughput, direct perchloric acid procedure coupled with a GC/MS stable isotope dilution technique was applied to measure ethanol and acetaldehyde concentrations 6, 36, 37. In brief, 250 µl of pre-chilled, 0.6 N perchloric acid solution containing internal standards, deuterated-ethanol, and deuterated-acetaldehyde, was added to 25 µl of blood, or 5 µl of bile, or about 25 mg of frozen tissue thawed over ice. The blood samples were then vortexed for 60 sec, and biles were vortexed for 30 sec followed by centrifugation at 13,000 g for 15 min at 4 °C. Solid tissues were then homogenized using Precellys® 24-homogenization Evolution Cryolys (Bertin Technologies, France) followed by the same centrifugation. Afterward, 200 µl of the supernatant was taken into a 20 mL glass vial for data acquisition by Agilent 8890 B GC/5977B MS through a 7697A headspace autosampler (Agilent Technologies, Santa Clara, CA). The concentrations of ethanol and acetaldehyde in samples were calculated by comparing the integrated areas of ethanol and acetaldehyde peaks on the gas chromatograms with those of internal standards added in each sample.

**Western blot:** Liver tissues used in this project were homogenized by using RIPA lysis buffer (1 mg tissue/60 μL) containing protease inhibitors (Santa Cruz, CA) following the manufacturer's instruction. Samples were processed on ice and then were centrifuged at 13,200g for 10 minutes at 4 °C in a microcentrifuge. The concentrations of protein extracts were determined by using BCA Protein Assay Kit (Thermo Fisher, Waltham, MA, USA), then were mixed with loading buffer, and loaded equal amounts of protein samples into the wells of 4-12% Bis-Tris protein gels (Bio-Rad, Hercules, CA, USA). Transferring the proteins to nitrocellulose membranes (Thermo Fisher, Waltham, MA, USA). Protein bands were visualized and analyzed by using SuperSignal West Femto Maximum Sensitivity Substrate (Thermo Fisher, Waltham, MA, USA). The results were determined by ImageJ software (National Institutes of Health, Bethesda, MD). The primary antibodies used for analysis include anti-PPAR-γ (1:1000; abcam, Cat. #: ab209350), anti-FAS (1:1000; abcam, Cat. #: ab128870), anti-CD163 (1:1000; abcam, Cat. #: ab182422), anti-HO-1 (1:1000, abcam, Cat. #: Ab189491) anti-β-Actin (1:10,000; Sigma-Aldrich, Cat. #: A1978). Secondary horseradish peroxidase-conjugated antibodies (1:5000; Cell Signaling Technology, Cat. #: 7076 (mouse) and 7074 (rabbit)) were used for analysis.

**Immunohistochemistry staining:** The sample preparation procedure and staining details remain the same as in a previous study 38. DAB Peroxidase Substrate Kit (Vector Laboratories, Inc.) was used to visualize the staining according to the manufacturer's instructions. The numbers of IBA and MPO^+^ cells were counted by ImageJ software (NIH).

**RNA isolation and real-time quantitative PCR (RT-qPCR):** Total RNA was extracted from the liver by using TRIzol reagent (Invitrogen, Carlsbad, CA) following the manufacturer's instruction. 1 μg total RNA was reversed transcribed into cDNA by using High-Capacity cDNA Reverse Transcription kit (Thermo Fisher Scientific). RT-qPCR was performed by SYBR Green Realtime PCR master mix (ABM, BlasTaq^TM^2XqPCR, Cat. #: G892). The mRNA levels were determined by QuantStudioTM 6 Real-Time PCR System (278861830; Thermo Fisher Scientific). The expression levels of target genes were normalized to 18S rRNA expression. Comparative Ct (2-ΔΔCt) method was performed to quantify the mRNA expression level. All primers are listed in **Supplementary Table 1**.

### Quantification and statistical analysis

Data was presented as the means ± SD or means ± SEM and were analyzed by using GraphPad Prism software (V.10; GraphPad Software, La Jolla, California, USA). To compare values from two groups, significance was evaluated by the student t-test. Data from multiple groups were compared with one-way ANOVA or two-way ANOVA analysis followed by Tukey's post hoc test. All statistical tests were two-sided. P values < 0.05 were considered statistically significant.

## Supplementary Material

Supplementary figures and table.
