# Supplementary material for: Hepatic Aquaporin 8 Promotes Alcohol Consumption and Ameliorates Alcohol-Induced Liver Injury by Facilitating Acetaldehyde Excretion
Source: Int J Biol Sci. 2026 Jan 1;22(2):566–81. doi: 10.7150/ijbs.122713 (PMC12780843; doi:10.7150/ijbs.122713)
Supplement: Supplementary file 1 — Supplementary figures and table. [file ijbsv22p0566s1.pdf]

Supplementary Figure Legends

Sup Fig 1.

A. Representative image of gallbladder catheterization under the microscope. Under microscopic visualization, the common bile duct was double-ligated proximal to the duodenum to obstruct endogenous bile drainage. A polyethylene (PE-5) catheter was carefully inserted into the gallbladder and secured with two ligatures to allow continuous bile flow for collection and measurement.

B. Detailed dynamic bile volume comparison at indicated time points in WT and *Aqp8* KO mice with or without EtOH gavage (n=7-8 per group)

Sup Fig 2.

A. Isolated primary hepatocytes were incubated with 1mM AcH for 5,15,30 mins. Hepatocytes and supernatant AcH levels, and hepatocytes/supernatant (Hep/Sup) AcH ratio at each time point of *Aqp8* KO and WT isolated primary hepatocytes (The experiment was repeated 4 times independently).

Sup Fig 3.

A. EtOH consumption in male and female WT and *Aqp8* KO mice at each percentage (n=9-10 per group).

B. Sweetness 2-Bottle Choice: saccharine liquid consumption and preference in WT (n=12) and *Aqp8* KO mice (n=11) with 2 saccharine concentrations. Values represent

means ± SD or SEM. *P<0.05, **P<0.01, ns=no significance. A two-sided Student's t-test was used for the comparison between the two groups.

C. D. WT and *Aqp8* KO received 5 g/kg EtOH binge (by gavage), and samples were collected 3/6/9 hours later. Liver, bile, and blood AcH levels were measured. (n=4-6)

Sup Fig 4.

A. Serum ALT levels in WT and *Aqp8* KO mice in two sexes with pair-fed or 5% EtOH diet.

B. The liver/body weight ratio in WT and *Aqp8* KO mice with pair-fed or 5% EtOH diet.

C. Food consumption in WT and *Aqp8* KO mice with pair-fed or 5% EtOH diet.

D. Liver *Aqp8* mRNA expression in WT and *Aqp8* KO mice with pair-fed or 5% EtOH diet.

E. Representative liver H&E staining in WT and *Aqp8* KO mice with pair-fed or 5% EtOH diet.

F. Representative IBA1, and MPO IHC staining and IBA and MPO positive cell counts in WT and *Aqp8* KO mice with 5% EtOH diet.

Sup Fig 5.

A. Food consumption in *Aqp8* KO GFP control mice (*Aqp8* KO $^{\text{Ad-GFP}}$) and liver AQP8 overexpression in *Aqp8* KO mice (*Aqp8* KO $^{\text{Ad-}\textit{Aqp8}}$) with 5% EtOH diet.

B. Serum ALT levels in *Aqp8* KO $^{\text{Ad-GFP}}$ and *Aqp8* KO $^{\text{Ad-}\textit{Aqp8}}$ mice.

C. Liver *Aqp8* mRNA expression in *Aqp8* KO $^{\text{Ad-GFP}}$ and *Aqp8* KO $^{\text{Ad-}\textit{Aqp8}}$ mice.

D. Bile volume in *Aqp8* KO $^{\text{Ad-GFP}}$ and *Aqp8* KO $^{\text{Ad-}\textit{Aqp8}}$ mice.

Sup Fig. 1

A

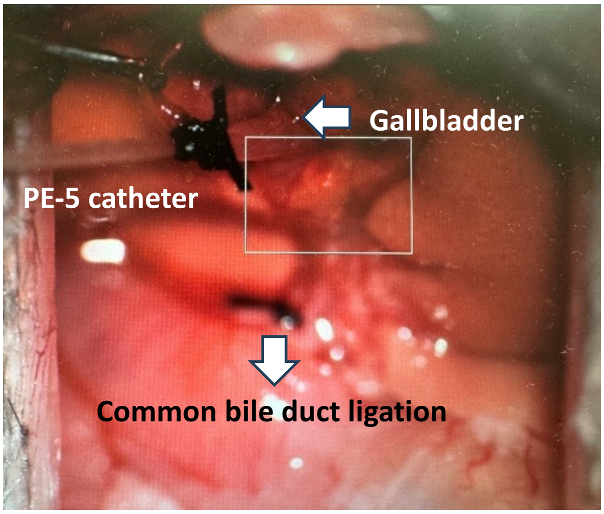
Gallbladder
PE-5 catheter
Common bile duct ligation

B

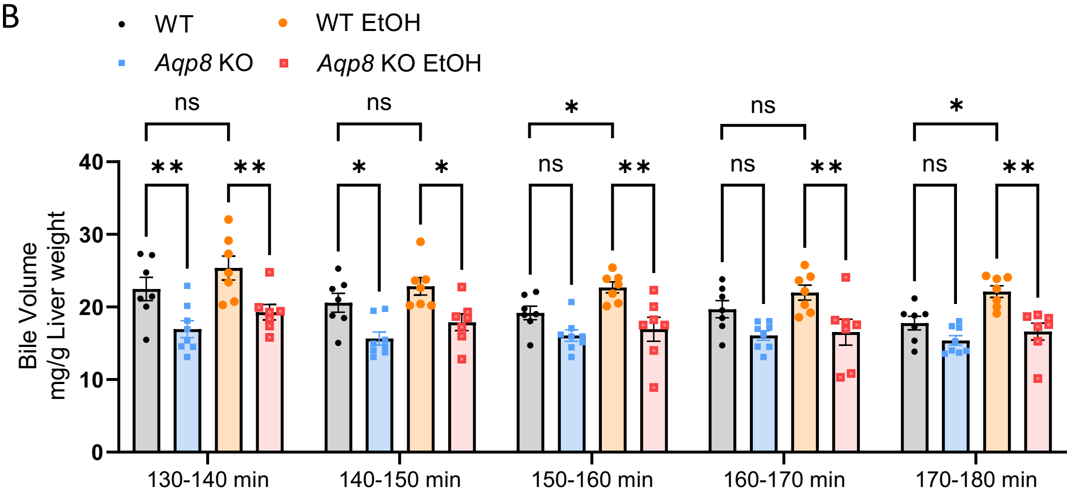
WT
WT EtOH
Aqp8 KO
Aqp8 KO EtOH
Bile Volume
mg/g Liver weight
0
10
20
30
40
ns
**
**
*
*
ns
**
ns
**
ns
**
*
ns
*
130-140 min
140-150 min
150-160 min
160-170 min
170-180 min

Sup Fig. 2

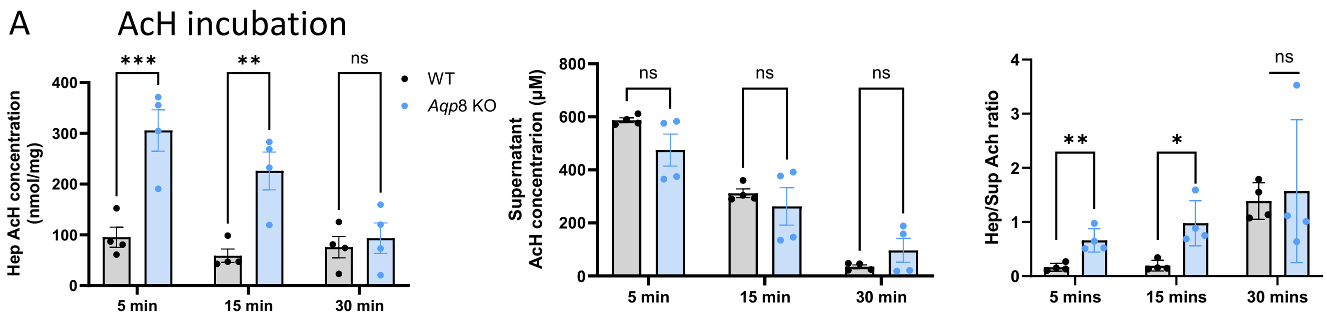
A
AcH incubation
Hep AcH concentration (nmol/mg)
400
300
200
100
0
***
**
ns
5 min
15 min
30 min
WT
Aqp8 KO
Supernatant AcH concentrarion (µM)
800
600
400
200
0
ns
ns
ns
5 min
15 min
30 min
Hep/Sup Ach ratio
4
3
2
1
0
**
*
ns
5 mins
15 mins
30 mins

Sup Fig. 3

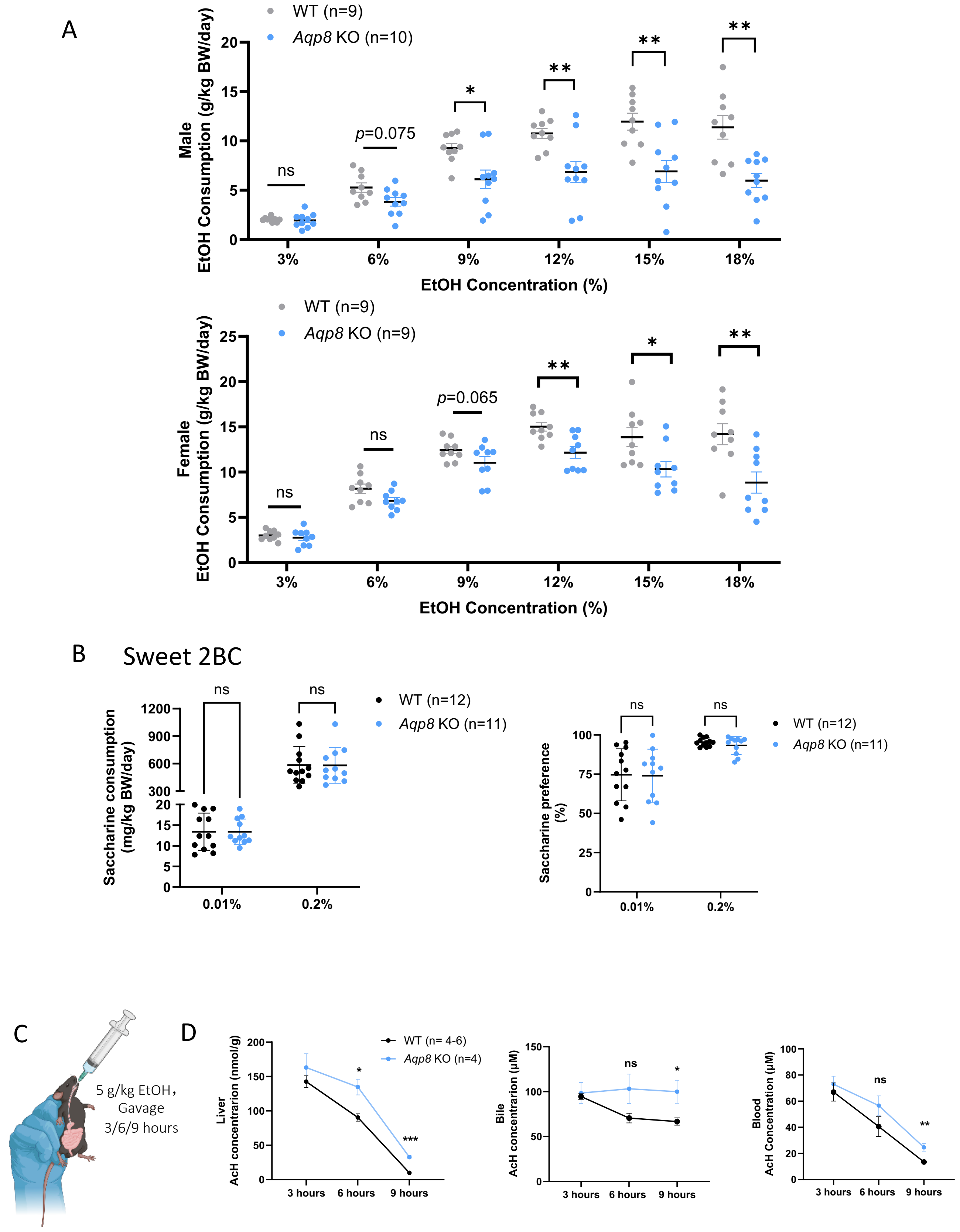

A
WT (n=9)
Aqp8 KO (n=10)
Male
EtOH Consumption (g/kg BW/day)
20
15
10
5
0
ns
p=0.075
*
**
**
**
3%
6%
9%
12%
15%
18%
EtOH Concentration (%)
WT (n=9)
Aqp8 KO (n=9)
Female
EtOH Consumption (g/kg BW/day)
25
20
15
10
5
0
ns
ns
p=0.065
**
*
**
3%
6%
9%
12%
15%
18%
EtOH Concentration (%)
B
Sweet 2BC
Saccharine consumption
(mg/kg BW/day)
1200
900
600
300
20
15
10
5
0
ns
ns
0.01%
0.2%
WT (n=12)
Aqp8 KO (n=11)
Saccharine preference
(%)
100
75
50
25
0
ns
ns
0.01%
0.2%
WT (n=12)
Aqp8 KO (n=11)
C
5 g/kg EtOH,
Gavage
3/6/9 hours
D
Liver
AcH concentrarion (nmol/g)
200
150
100
50
0
WT (n= 4-6)
Aqp8 KO (n=4)
*
***
3 hours
6 hours
9 hours
Bile
AcH concentrarion (μM)
150
100
50
0
ns
*
3 hours
6 hours
9 hours
Blood
AcH Concentration (μM)
100
80
60
40
20
0
ns
**
3 hours
6 hours
9 hours

Sup Fig. 4

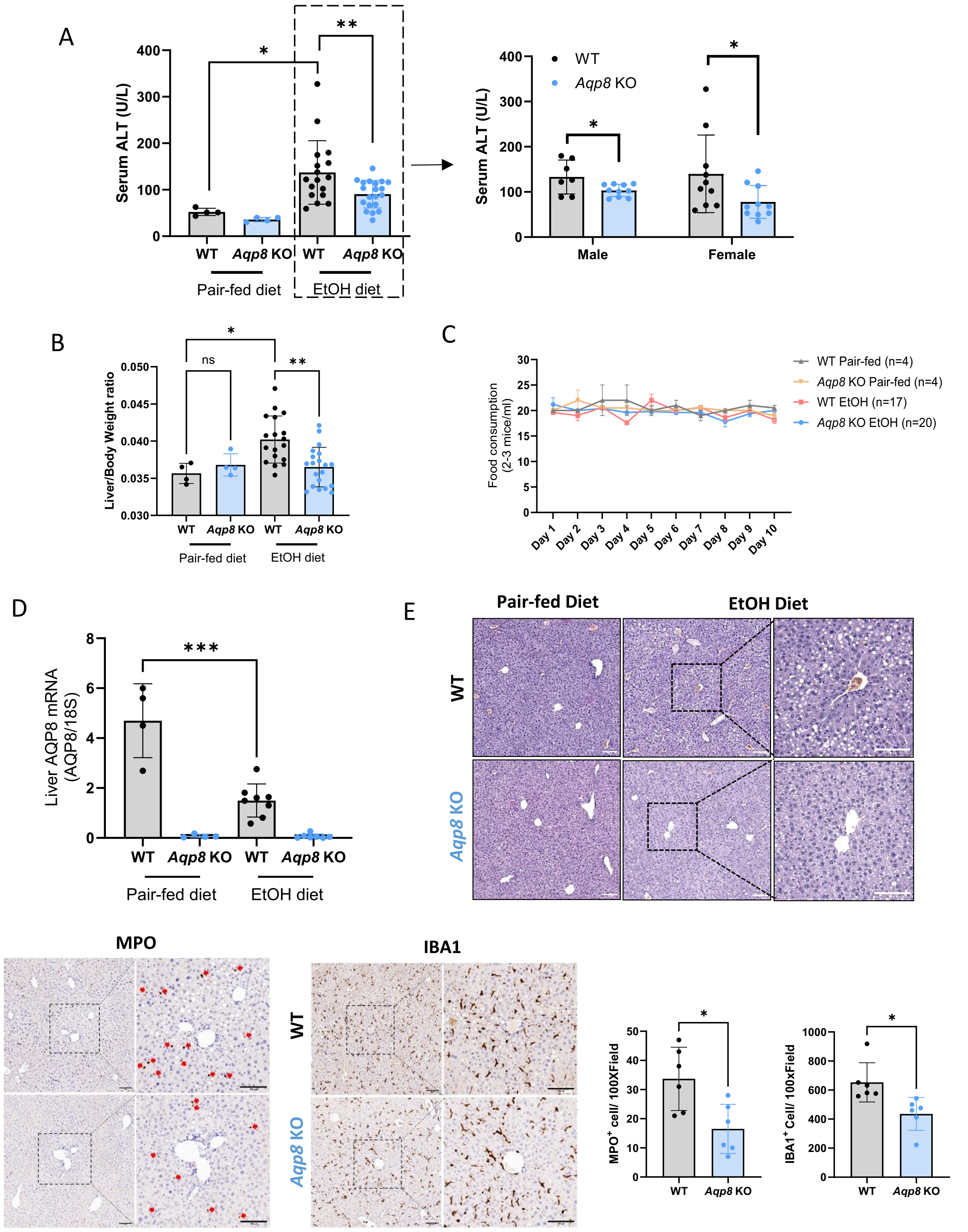

A
Serum ALT (U/L)
WT
Aqp8 KO
Pair-fed diet
EtOH diet
Male
Female
B
Liver/Body Weight ratio
ns
C
Food consumption (2-3 mice/ml)
WT Pair-fed (n=4)
Aqp8 KO Pair-fed (n=4)
WT EtOH (n=17)
Aqp8 KO EtOH (n=20)
Day 1
Day 2
Day 3
Day 4
Day 5
Day 6
Day 7
Day 8
Day 9
Day 10
D
Liver AQP8 mRNA (AQP8/18S)
E
Pair-fed Diet
EtOH Diet
F
MPO
IBA1
MPO+ cell/ 100XField
IBA1+ Cell/ 100xField

Sup Fig. 5

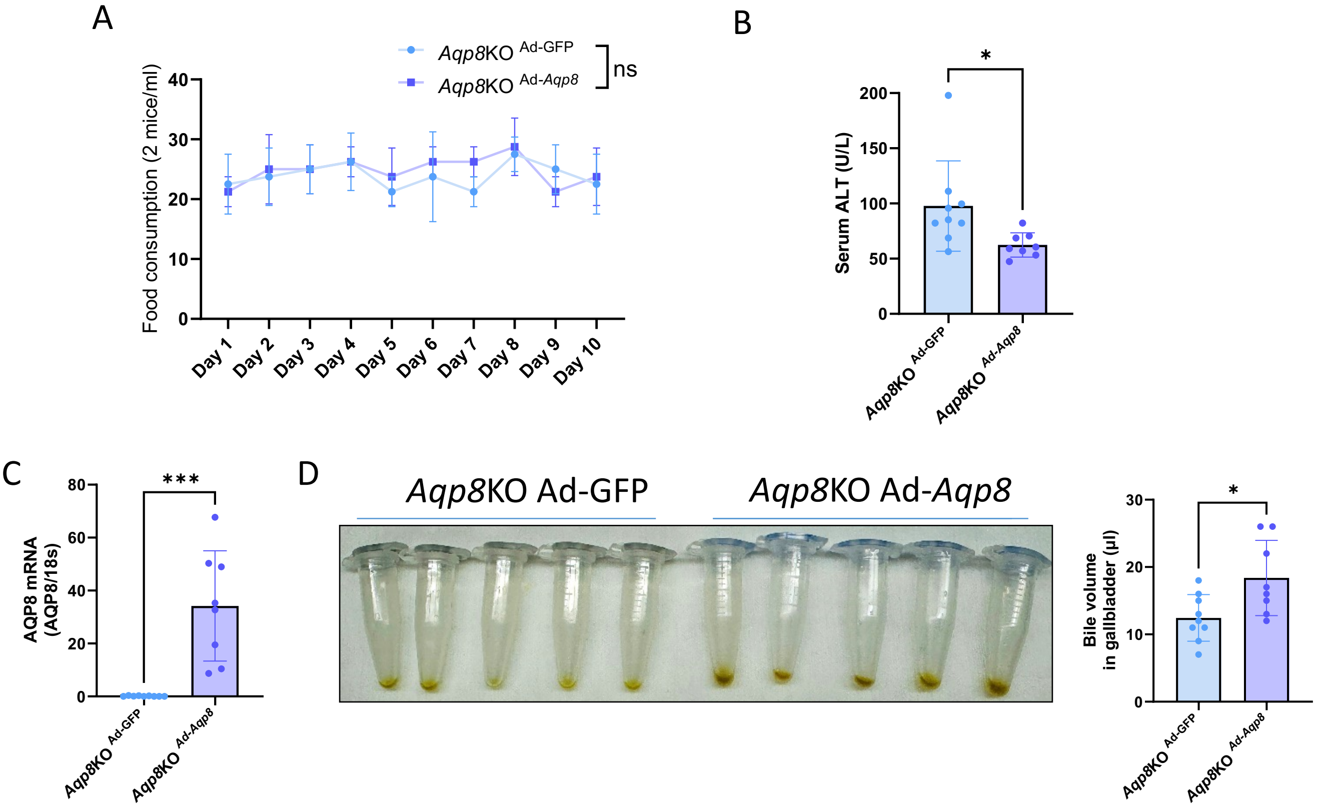

A
Aqp8KO Ad-GFP
Aqp8KO Ad-Aqp8
ns
Food consumption (2 mice/ml)
40
30
20
10
0
Day 1
Day 2
Day 3
Day 4
Day 5
Day 6
Day 7
Day 8
Day 9
Day 10
B
*
Serum ALT (U/L)
200
150
100
50
0
Aqp8KO Ad-GFP
Aqp8KO Ad-Aqp8
C
***
AQP8 mRNA (AQP8/18s)
80
60
40
20
0
Aqp8KO Ad-GFP
Aqp8KO Ad-Aqp8
D
Aqp8KO Ad-GFP
Aqp8KO Ad-Aqp8
*
Bile volume in gallbladder (µl)
30
20
10
0
Aqp8KO Ad-GFP
Aqp8KO Ad-Aqp8

Sup Table 1. Primer list

Gene	Forward Primer (5’-3’)	Reverse Primer (5’-3’)
Aqp8	aacatcagcggtggacactt	cagccctccaaatagctggg
Lxr-β	ctcccacccacgcttacac	gccctaacctctctccactca
Abcg1	gctgtgcgttttgtgctgtt	tgcagctccaatcagtagtcctaa
Fas	attgcatcaagcaagtgcag	gagccgtcaaacaggaagag
Cd163	tgggtggggaaagcataact	aagttgtcgtcacacaccgt
Scd-1	ttcttgcgatacactctggtgc	cgggattgaatgttcttgtcgt
Il-1β	tcgctcagggtcacaagaaa	ctcagaggcaaggaggaaac
Il-10	atcgatttctcccctgtgaa	tgtcaaattcattcatggcct
Ccl5	gtgcccacgtcaaggagtat	ccacttcttctctgggttgg
Ho-1	aggctaagaccgccttcct	tgtgttcctctgtcagcatca
Il-6	acaagtcggaggcttaattacacat	ttgccattgcacaactcttttc
Srebp1c	ggcccgggaagtcactgt	ggagccatggattgcacatt
Sod-1	tactgatggacgtggaaccc	gaaccatccacttcgagca
18s	acggaagggcaccaccagga	caccaccacccacggaatcg
Ccl2	ttaaaaacctggatcggaaccaa	gcattagcttcagatttacgggt
Ccl20	tctggacctcaaaatcctgc	tggagtagcttcttcaccca
